# Cellular Prion Protein Is Closely Associated with Early Recurrence and Poor Survival in Patients with Hepatocellular Carcinoma

**DOI:** 10.3390/diagnostics12071635

**Published:** 2022-07-05

**Authors:** Mo-Jong Kim, Yoon-Ah Cho, Eunhye Kim, Ji-Young Choe, Ji-Won Park, Junyong Lee, Jung-Woo Lee, Sung-Hoon Moon, Yong-Sun Kim, Sung-Eun Kim, Eun-Kyoung Choi

**Affiliations:** 1 Ilsong Institute of Life Science, Hallym University, Seoul 07247, Korea; hanbami0730@gmail.com (M.-J.K.); yskim@hallym.ac.kr (Y.-S.K.); 2 Department of Biomedical Gerontology, Graduate School, Hallym University, Chunchoen 24252, Korea; 3 Department of Pathology, Hallym University Sacred Heart Hospital, College of Medicine, Hallym University, Anyang 14068, Korea; purpleice21@hallym.or.kr; 4 Department of Internal Medicine, Hallym University Sacred Heart Hospital, College of Medicine, Hallym University, Anyang 14068, Korea; eunhye94@hallym.or.kr (E.K.); miunorijw@hallym.or.kr (J.-W.P.); endomoon@hallym.or.kr (S.-H.M.); 5 Anatomic Pathology Reference Lab, Seegene Medical Foundation, Suwon 16580, Korea; hury9210@naver.com; 6 Institute for Liver and Digestive Diseases, Hallym University, Chuncheon 24252, Korea; 7 Department of Anesthesiology and Pain Medicine, Seoul Medical Center, Seoul 02053, Korea; junyo.lee@seoulmc.or.kr; 8 Department of Surgery, Hallym University Sacred Heart Hospital, College of Medicine, Hallym University, Anyang 14068, Korea; km98woo@hallym.or.kr; 9 Department of Microbiology, College of Medicine, Hallym University, Chuncheon 24252, Korea

**Keywords:** prion protein, hepatocellular carcinoma, recurrence, survival, hepatic resection

## Abstract

The cellular prion protein (PrP^C^) is known to play a role in cancer proliferation and metastasis. However, the role of PrP^C^ expression in hepatocellular carcinoma (HCC) is unknown. This study investigated whether overexpression of PrP^C^ affects recurrence after surgical resection and survival in HCC. A total of 110 HCC patients who underwent hepatic resection were included. They were followed up for a median of 42 months (range 1–213 months) after hepatectomy. The relationships between PrP^C^ expression and the HCC histologic features, recurrence of HCC following surgical resection, and survival of the patients were examined. Seventy-one cases (64.5%) of HCC demonstrated higher expression of PrP^C^. The expression of PrP^C^ was only correlated with diabetes mellitus. There was no association between PrP^C^ expression and age, sex, hypertension, hepatitis B virus positivity, alcohol consumption, Child–Pugh class, major portal vein invasion, serum alpha-fetoprotein, and HCC size or number. The 1-year recurrence rates in patients with higher PrP^C^ expression were higher than those with lower PrP^C^ expression. The cumulative survival rates of patients with higher PrP^C^ expression were significantly shorter than those of patients with lower PrP^C^ expression. In conclusion, PrP^C^ expression is closely associated with early recurrence and poor survival of HCC patients following surgical resection.

## 1. Introduction

Liver cancer is known the fifth most common cancer and the second leading cause of cancer-related mortality in the world. Hepatocellular carcinoma (HCC) accounts for approximately 90% of liver cancers [1,2,3,4]. Surgical treatment for HCC, such as resection and liver transplantation, and potentially curative treatments are applied to 30–40% of patients in Western counties and a smaller proportion of patients in Asia [2]. In terms of therapeutic aspects, HCC has several characteristics that are different from those of other cancers. First, because 90% of HCC patients have comorbidities such as chronic hepatitis B, chronic hepatitis C, or cirrhosis, curative treatment is often difficult due to poor underlying liver function, and the risk of recurrence continues even after 5 years or more after treatment. In particular, the 5-year recurrence rate of liver transplantation or surgical resection, which can expect the best prognosis thus far in HCC treatment, reached 8–20% and 50–70%, respectively [2]. Although hepatic resection is the treatment of choice in HCC patients with non-cirrhotic liver, 5% of these patients in the West and 40% in Asia are eligible for surgery [5]. With these results, even in HCC patients who undergo surgical treatments, frequent recurrence after surgical surgery is a major limitation to long-term survival [6]. In addition, HCC recurrence complicates 70% of cases at 5 years, reflecting either intrahepatic metastases or the development of de novo tumors [1]. Beyond surgical treatments, there are other locoregional therapies such as radiofrequency ablation and trans-arterial chemoembolization, etc. There were no effective systemic anticancer agents for HCC therapy until sorafenib, an oral multityrosine kinase inhibitor, was approved in 2007 [7,8]. Although sorafenib-treated HCC patients showed a median survival of 10.7–13.4 months compared to untreated advanced-stage HCC patients, with a median survival of ~8 months [7,9,10], it was not considered to have sufficient effect in terms of the treatment of HCC. Recently, other systemic therapies, including immune checkpoint inhibitors, tyrosine kinase inhibitors, and monoclonal antibodies, have challenged the use of conventional therapies for HCC [11]. However, there is still needed to develop new anticancer drugs, and enhancing the effects of existing anticancer drugs should be explored. Therefore, the discovery of biomarkers predicting the recurrence and survival of HCC is still a major issue in many studies.

Cellular prion protein (PrP^C^) is a glycosylphosphatidylinositol (GPI)-anchored protein, and it was proposed that misfolding PrP^C^ plays an important key in neurodegenerative diseases [12,13]. The PrP^C^ has important roles in regulating cellular processes such as cell proliferation, differentiation, survival, and death in the nervous and immune systems [14,15]. Interestingly, several studies have shown that PrP^C^ expression is associated with cancer proliferation, metastasis, and drug resistance in various cancers [13,16]. Although the expression level of PrP^C^ is very low in the liver as a nonneuronal tissue compared to neuronal tissues, it has been demonstrated that PrP^C^ expression is age-dependent and is higher in liver tissue of females compared to males [17]. In addition, upregulation of PrP^C^ expression under oxidative stress has been shown in activated hepatic stellate cells [18], and the number of PrP^C^-positive cells was associated with disease activity in patients with chronic hepatitis B, chronic hepatitis C, autoimmune hepatitis, or alcoholic liver disease [19]. Interestingly, Yang et al. demonstrated the overexpression of PrP^C^ in human HCC tissue although PrP^C^ expression has no diagnostic or prognostic role [20]. This study aimed to investigate whether expression of PrP^C^ in human HCC tissues affects the recurrence and survival in HCC patients treated with surgical resection.

## 2. Materials and Methods

### 2.1. Patients and Tissue Microarray

A total of 110 patients treated with surgical resection as first-line treatment for HCC from January 2011 to December 2015 at Hallym University Sacred Heart Hospital (Anyang, Korea) were enrolled. The patients were followed-up for a median period of 42 months (range 1–213 months) after hepatectomy. Recurrence and survival were determined using medical records at the last follow-up date. Patients who fulfilled all of the inclusion criteria were included: (1) no previous treatment for HCC before surgery; (2) histologic confirmation of HCC; (3) complete tumor resection with negative margins; (4) a single lesion smaller than 8 cm; and (5) up to three lesions, each smaller than 3 cm. After surgery, patients were followed-up once a month for the first months and then every 3 months thereafter. Serum alpha-fetoprotein (AFP) and liver function tests were performed routinely at the postoperative visits. Computed tomography (CT) or magnetic resonance imaging (MRI) was performed every 3 months. Once tumor recurrence was confirmed by these examinations, patients received optimal treatment. Patient death was determined from death certificates or phone follow-up. 

Tissue microarrays (TMAs) were constructed in which a 2 mm sized tissue core was perforated and re-embedded from the labeled area. Each sample was arrayed in duplicate to minimize tissue loss. Informed consent was waived because this study was retrospective and analyzed anonymous clinical data. This study was approved by the ethics committee of Hallym University Sacred Heart Hospital. The study was conducted with the approval of the Institutional Review Board of Hallym University Sacred Heart Hospital (HALLYM 2022-03-012-001). 

### 2.2. Immunohistochemical Staining of PrP^C^ in a Human HCC Tissue Microarray

Tissue microarray blocks were cut into 4 µm thick slices, and the sections were used for immunohistochemical staining. Briefly, TMA slices were deparaffinized with xylene, hydrated in a graded ethanol series, and then treated with peroxidase-blocking solution (Dako, Copenhagen, Denmark) for 10 min to block endogenous peroxidase, as previously described [21]. The sections were blocked with protein block solution (Dako) for 60 min at room temperature (RT) and then incubated with rabbit polyclonal antibody-PrP (8H4; Sigma-Aldrich, St. Louis, MO, USA) diluted antibody diluent. After primary antibody incubation, the sections were detected using the Dako EnVision Detection System kit (Dako) according to the manufacturer’s instructions.

### 2.3. Evaluation of Immunostaining

Slides of the TMA were examined the proportion of tumor cells that were positive for PrP^C^ in the tumor cell nuclei. For each spot, areas of the most intense and/or predominant staining patterns were scored. Their staining intensity was usually positively correlated with the proportion of positive tumor cells. To analyze the TMA, slices were scored semi-quantitatively using a four-graded system based on the intensity and distribution similar to previous study [21]: graded 0, undetectable; graded 1, weak staining, graded 2; medium staining, graded 3; strong staining. All TMAs were examined by light microscopy (BX51; Olympus, UK). Based on the findings, all TMAs were classified and the clinical data effectively analyzed: (1) a PrP^C^-low group (less than 50%, none, or grade (1)) and (2) a PrP^C^-high group (over 50%, grade 2, or grade (3)). All tissue samples were reviewed by two experienced pathologists (Cho Y.A., Choe J.Y.). Two independent observers determined PrP^C^ expression levels using the arrays, and both pathologists re-examined specimens with discrepant scores to determine a consensus score.

### 2.4. Statistical Analysis 

To avoid confusion of analysis and focus on the role of PrP^C^ as a prognostic factor in HCC recurrence and overall survival, we applied the following standards in this study. First, we considered only when HCC recurrence was observed according to radiologic evidence. Second, only deaths from hepatic origin were considered for overall survival. We did not analyze non-hepatic origin deaths in determining cumulative survival rates. Based on the same criteria, multivariate analyses for recurrence and survival were performed using the Cox regression hazard model or logistic regression model.

Data are expressed as the mean ± SD or median (range). For statistical significance, Student’s *t*-test and χ^2^ test were conducted for comparisons of variables between groups. The cumulative recurrence and survival were evaluated by the Kaplan–Meier method, and differences were determined by the log rank test. A multivariate analysis was carried out to identify independent predictors for recurrence and survival using a Cox regression hazard model or logistic regression model. A value of *p* < 0.05 was regarded as significant. All analyses were performed using SPSS 27.0 software (SPSS, Inc., Chicago, IL, USA) and GraphPad Prism 9 program (GraphPad software, San Diego, CA, USA).

## 3. Results

### 3.1. Baseline Characteristics of Enrolled Patients

Table 1 showed baseline characteristics according to PrP^C^ expression in enrolled patients. Sixty-nine patients (44.5%) had underlying liver cirrhosis. The majority of the patients (92.7%, 101/109) had relatively well-reserved liver function with Child–Pugh class A. A total of 85 patients had HCC less than 5 cm in diameter, and 25 had HCC more than 5 cm in diameter. The mean tumor size was 3.56 ± 1.84, and the mean tumor number was 1.24 ± 0.54. The baseline characteristics of the 110 patients are listed in Table 1.

### 3.2. Differences According to PrP^C^ Expression

**Figure 1:**
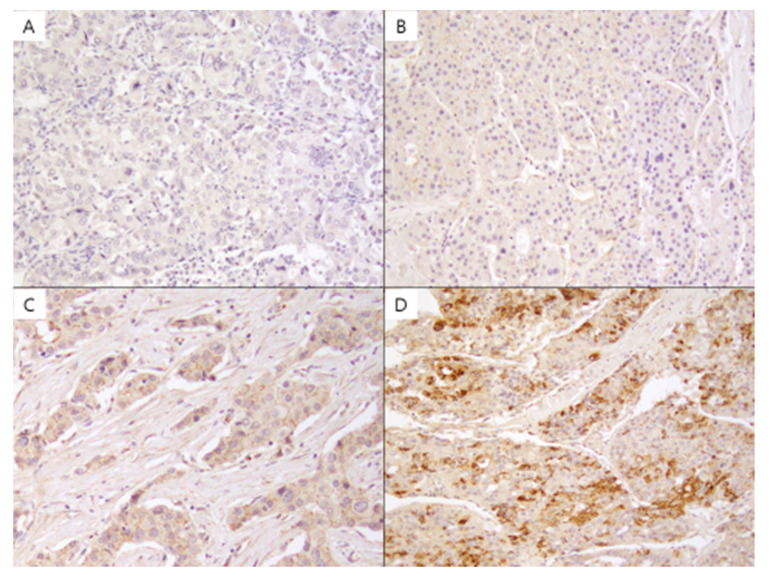
PrP^C^ was immunohistochemically stained, and its intensity was graded as none (**A**), + (**B**), ++ (**C**), and +++ (**D**). Original magnification ×20.

Of the 110 HCC samples, PrP^C^ was stained in 109 (99.1%), but only 5 of the surrounding liver tissues were stained. Of 109 positive samples, 38 were grade 1, 69 were grade 2, and 2 samples showed grade 3 expression (Figure 1). The proportion of patients with high-PrP^c^ staining (grade 2 or grade 3) was 71.8% (79/110) compared to 35.5% (39/110) with low-PrP^c^ staining (0 or grade 1). To explore the clinical significance of PrP^C^ in HCC, the association between the expression level of PrP^C^ and the clinical features of patients with HCC was analyzed. Patients with low PrP^C^ expression had more diabetes mellitus (DM) (*p* = 0.033). However, the expression of PrP^C^ did not show significant differences with age, sex, hepatitis B surface antigen (HBsAg) positivity, hypertension, liver cirrhosis, liver function, tumor size, tumor number, AFP, and vascular invasion, as shown in Table 1.

### 3.3. Association of PrP^C^ Expression with Survival in Patients with HCC

**Figure 2:**
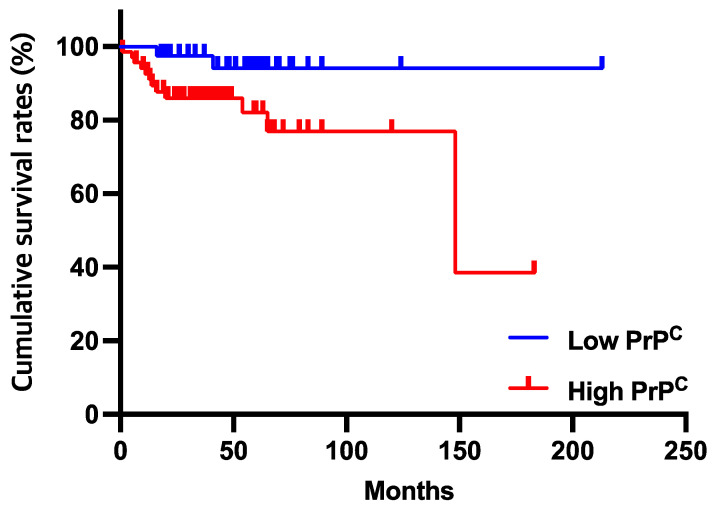
The cumulative survival rates in HCC patients with high PrP^C^ were significantly lower than those of patients with low PrP^C^ (*p* = 0.043). The patients with high PrP^C^ showed cumulative survival rates of 93%, 87%, and 85% at 1, 3, and 5 years, respectively. However, the proportion of patients with low PrP^C^ were 95%, 95%, and 95%, respectively.

To determine the effect of PrP^C^ expression on overall survival in patients with HCC, we conducted Kaplan–Meier analysis to compare the survival between patients with high PrP^C^ expression and low PrP^C^ expression. The 1-, 3-, and 5-year cumulative survival rates of patients with high PrPC expression were significantly shorter than those of patients with low PrP^C^ expression (93%, 87%, and 85% vs. 95%, 95%, and 95%, respectively; *p* = 0.043). (Figure 2).

### 3.4. Univariate and Multivariate Analysis of Overall Survival

The overall survival of HCC patients with low PrP^C^ expression was significantly shorter than that of patients with high PrP^C^ expression. To evaluate associated factors for patient survival, univariate and multivariate Cox regression analyses were conducted. Univariate analysis demonstrated that larger tumor size (≥5 cm) and high PrP^C^ expression were potential candidates for multivariate analysis of survival (*p* < 0.1 on univariate analysis). On multivariate analysis, larger tumors and high PrP^C^ expression were independent risk factors for postoperative survival (Table 2).

**Figure 3:**
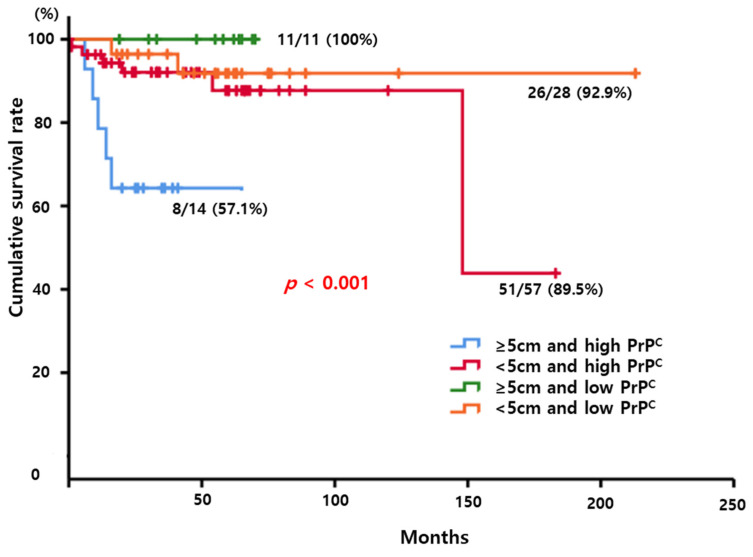
Kaplan–Meier curve to compare the cumulative survival rate according to four combinations between tumor size and PrP^C^ expression. HCC patients with larger tumor and high PrP^C^ expression had poor cumulative survival (*p* < 0.001).

To evaluate synergistic effect between tumor size and PrP^C^ expression, we performed Kaplan–Meier analysis to compare the cumulative survival rate according to four combinations between tumor size and PrP^C^ expression. Definitely, HCC patients with high PrP^C^ expression and large tumor size showed poor survival. The cumulative survival rates of HCC patients with ≥5 cm sized tumor and high PrP^C^ expression, <5 cm sized tumor and high PrP^C^ expression, ≥5 cm sized tumor and low PrP^C^ expression, and <5 cm sized tumor and high PrP^C^ expression had statistically significant difference (57.1%, 89.5%, 92.9%, and 100%, respectively; *p* < 0.001). Cox regression analyses demonstrated that HCC patients with larger tumor size (≥5 cm) and high PrP^C^ expression showed hazard ratio (HR) 11.2 with 95% confidence interval (CI) (2.155–58.825, *p* = 0.004). (Figure 3)

### 3.5. Association of PrP^C^ Expression with Recurrence in Patients with HCC 

**Figure 4:**
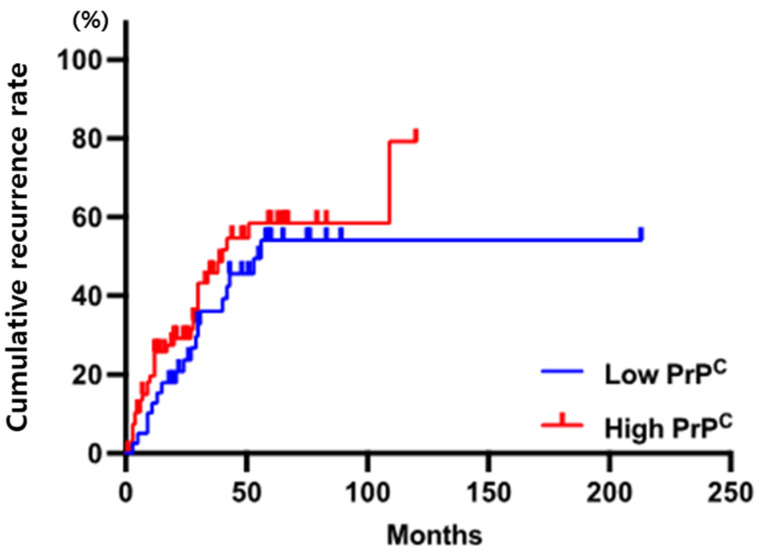
The cumulative recurrence rates in HCC patients with high PrP^C^ were higher than those of patients with low PrP^C^. The patients with high PrP^C^ showed cumulative survival rates of 23.9%, 36.6%, and 42.3% at 1, 3, and 5 years, respectively. However, the proportion of patients with low PrP^C^ were 20.5%, 23.1%, and 46.2%, respectively (*p* = 0.340).

To determine the role of PrP^C^ expression in the postoperative recurrence of patients with HCC, we performed Kaplan–Meier analysis to compare the cumulative recurrence rate between patients with high PrP^C^ expression and low PrP^C^ expression. The 1-, 3-, and 5-year cumulative recurrence rates of patients with high PrP^C^ expression demonstrated a higher tendency than those of patients with low PrP^C^ expression but did not show a significant difference (23.9%, 36.6%, and 42.3% vs. 20.5%, 23.1%, and 46.2%, respectively; *p* = 0.340) (Figure 4). Interestingly, we found a significant difference in 1-year recurrence after surgery: the 1-year recurrence of patients with high PrP^C^ expression was significantly higher than that of patients with low PrP^C^ expression using a logistic regression model (odds ratio (OR) 3.053, 95% CI 1.052–8.857, *p* = 0.04).

### 3.6. Univariate and Multivariate Analysis of 1-Year Recurrence Following Surgical Reaction

To evaluate associated factors for the 1-year recurrence in patients who underwent surgical resection, univariate and multivariate logistic regression analyses were conducted. Univariate analysis demonstrated that hypertension, larger tumor size (≥5 cm), higher AFP level (≥400 ng/mL), and high PrP^C^ expression were potential candidates for multivariate analysis of survival (*p* < 0.1 on univariate analysis). On multivariate analysis, larger tumors (OR 3.212, 95% CI 1.043–9.895, *p* = 0.042) and high PrP^C^ expression (OR 3.540, 95% CI 1.119–11.201, *p* = 0.031) were independent risk factors for postoperative survival (Table 3).

**Figure 5:**
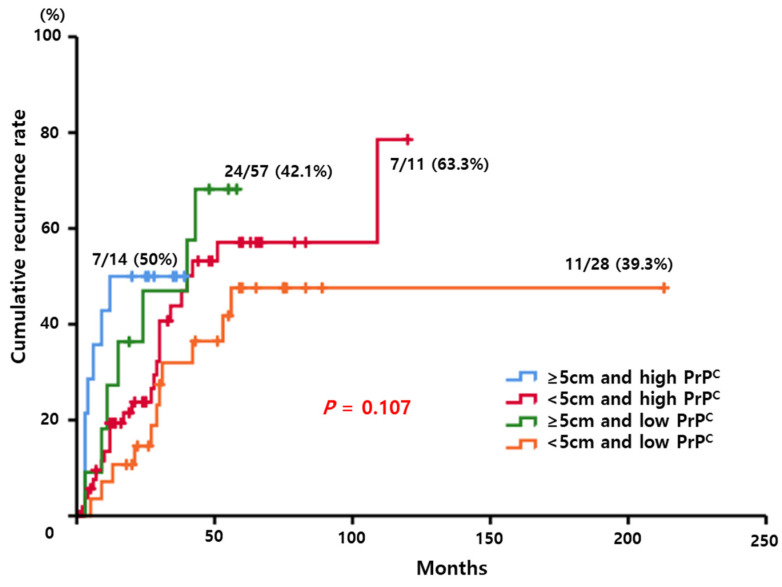
Kaplan–Meier curve to compare the cumulative recurrence rate according to four combinations between tumor size and PrP^C^ expression. HCC patients with larger tumor and high PrP^C^ expression had a higher tendency for recurrence (*p* = 0.107).

To evaluate synergistic effect between tumor size and PrP^C^ expression, we performed Kaplan–Meier analysis to compare the cumulative recurrence rate according to four combinations between tumor size and PrP^C^ expression. Although there were not statistically significance for recurrence of HCC (*p* = 0.107), HCC patients with high PrP^C^ expression and large tumor size showed relatively higher recurrence rate compared to other groups. Especially, in terms of short-term recurrence, HCC patients with high PrP^C^ expression and large tumor size showed OR 13 with 95% CI 2.194–77.037 in 1-year recurrence (*p* = 0.005). (Figure 5)

## 4. Discussion

The role and function of PrP^C^ in the proliferation, apoptosis, invasion, metastasis, drug resistance, and cancer stem cell (CSC) properties of different cancer types suggest that it is a promising therapeutic target for cancer treatment [13,22]. In gastric cancer, PrP^C^ promotes the proliferation and metastasis of cancer cells [23], and the expression of PrP^C^ is higher in metastatic gastric cancer than in nonmetastatic gastric cancer [24]. In addition, the expression of PrP^C^ increased the proliferation and migration of pancreatic ductal adenocarcinoma [25], colon cancer [26], and melanoma [27].

Recently, regulation of PrP^C^ by hypoxia appeared to be a major topic in the cancer research field [28]. Hypoxia is an important inducer of angiogenesis, and vascularization of the hypoxic area is necessary for further cancer development. Several studies have demonstrated that hypoxia-inducible factor-1 alpha (HIF-1α) expression increases in various cancers and plays an important role in cancer cell progression [29]. HIF-1α regulates the expression of various genes related to cancer cells proliferation and apoptosis, angiogenesis, metastasis, cancer metabolism, CSC maintenance, and chemoresistance to various cancers [30,31]. Under hypoxic conditions, PrP^C^ leads to cancer progression in colon cancer by targeting the HSP 70 member 1-like (HSPA1L)/HIF-1α/glycoprotein 78 (GP78) axis [32]. PrP^C^ was degraded via the proteasome pathway mediated by GP78, which is an ER membrane-anchored E3 ligase that regulates the progression of cancer cells. HIF-1α or HSPA1 L knockdown in a xenograft model inhibited cancer proliferation and liver metastasis [32]. Lee et al. also reported that patients with colon cancer had a significant association between high PrP^C^ expression and clinicopathological features such as advanced cancer stage, metastasis, and survival rate [32]. Recently, Yun et al. demonstrated that PrP^C^-positive cells had increased CSC properties. In PrP^C^-positive cells, the gene expression levels of CSC markers, metastasis, and angiogenesis were significantly upregulated; however, tumor suppressor gene expression was downregulated [33]. Exosomes induced by hypoxic drug-resistant colon cancer cells through upregulation of PrP^C^ expression increased sphere formation, invasion, migration, and proliferation of colon cancer. Anti-PrP^C^ antibody reduced the tumor size and serum PrP^C^ concentration in a xenograft model of colon cancer. In addition, 5-fluorouracil with an anti-PrP^C^ antibody reduced tumor size, tumor proliferation, and PrP^C^ expression in a colon cancer model [33]. These findings suggest that PrP^C^ could be a feasible cancer marker and therapeutic target.

In terms of solid tumors, the concept of hypoxia is essential in the treatment of HCC because hypoxia drives vascular endothelial growth factor (VEGF) production and angiogenesis through HIF-1α activation [34,35]. Recent studies have demonstrated that HIF-1α and HIF2α are major indicators of the poor prognosis of HCC patients. The role of HIF-2α depends especially on the cellular context [36]. Therefore, HIFs have been recognized as potential targets for HCC therapy.

This study demonstrated for the first time that the expression of PrP^C^ was closely associated with short-term recurrence and survival in HCC patients who underwent surgical resection. Although 99.1% of HCC showed PrP^C^ expression, there were different outcomes according to the degree of PrP^C^ staining. There was a statistical significance between PrP^C^ expression and DM. Although several studies demonstrated associations with PrP^C^ and DM [37,38,39], there have been no reports that the expression of PrP^C^ in HCC tissues is related to DM. Oskarsson et al. demonstrated that intravenous injection of synthetic islet amyloid polypeptide (IAPP) aggregates can accelerate IAPP aggregate formation in islets in a rodent model of type 2 DM [40]. The amount of IAPP injected is substantially higher than the physiological level of IAPP found in the blood; however, they suggest that blood can be an effective route for spreading of IAPP from islet to islet. Jackson et al. also suggested that IAPP aggregates might be present in the circulation because IAPP deposits positively stained with Congo red were found in blood vessels of the brain from individuals affected by T2D [41]. To confirm the association of PrP^C^ expression and DM, it is necessary to investigate circulating PrP^C^ and liver or pancreatic tissue in DM patients.

Interestingly, disease-free survival rates after surgical resection were significantly lower in patients with low PrP^C^ expression, and the 1-year recurrence rate of HCC after surgical resection was also significantly lower in patients with low PrP^C^ expression. Therefore, we suggest that the PrP^C^ expression level might be a prognostic indicator after curative hepatectomy for HCC. In this study, there was no association between PrP^C^ expression and age, sex, liver cirrhosis, underlying liver function, HBV positivity, tumor size, tumor number, or AFP level. Kitada et al. reported that PrP^C^ was positively stained in patients with chronic liver disease, while PrP^C^ was negatively stained in normal liver samples. In addition, they demonstrated that the number of PrP^C^-positive cells correlated with disease activity but not with the degree of fibrosis [19]. Therefore, the degree of PrP^C^ expression may not show a direct correlation with underlying liver function or cirrhosis. The present study revealed that larger tumors and high PrP^C^ expression were independent risk factors for postoperative survival in HCC patients. In addition, analyses for synergistic effect according to the four combinations between tumor size and PrPC expression demonstrated that HCC patients with ≥5 cm sized HCC and high PrPC expression had highest risk for mortality. The group with larger tumor size and high PrPC expression was revealed to have a major risk factor for short-term recurrence after surgical treatment. Because the number of subjects in this study was relatively small, more patients are needed to confirm the association between PrP^C^ expression and disease activity and fibrosis stage in liver diseases. This study suggests that the expression of PrP^C^ in human HCC tissue might have diagnostic or prognostic roles. 

The present study has some limitations. First, because the number of enrolled patients was relatively small, and only HCC patients who underwent surgery were targeted, it is not sufficient to confirm the role of PrP^C^ in each stage of HCC and the role of PrP^C^ chemoresistance in HCC. Second, there was inherent bias in the design because it was a retrospective study. Therefore, these findings need to be confirmed in large prospective studies. Third, we did not experimentally investigate the pathophysiologic mechanism of PrP^C^ in HCC. Fourth, PrP^C^ is widely expressed in the whole body, but PrP^C^ expression is relatively low in liver tissue [18,42] compared to neuronal tissues, which have highest abundance PrP^C^ [15]. Therefore, the investigation of the expression of PrP^C^ in blood or other body fluid is also warranted in HCC patients.

Molecular targeted therapies have changed the landscape of cancer treatment. Molecular subclasses HCC have been proposed as proliferation/nonproliferation, and the key drivers of HCC have been identified after sequencing more than 1500 samples in several studies [43]. To date, no specific biomarkers have been demonstrated to predict chemoresistance, HCC recurrence, or mortality for precision medicine in HCC. It is still an important task to find therapeutic target substances in HCC. In terms of new and promising therapeutic targets, studies on PrP^C^ in HCC are needed.

## 5. Conclusions 

PrP^C^ expression is closely associated with 1-year recurrence and poor survival in HCC patients underwent surgical resection. Therefore, the expression level of PrP^C^ in HCC tissues might be an important prognostic marker after curative surgery. More advanced and large research is warranted; however, this study suggests the possibility of the clinical application of anti-PrP^C^ antibody as an anticancer drug in HCC treatment.

## Figures and Tables

**Table 1:** Clinical characteristics according to PrP^C^ expression.

Variables	Total (*n* = 110)	Low (*n* = 39)	High (*n* = 71)	*p*-Value
Age (years)	62.35 ± 11.71	62 ± 9.97	62.5 ± 12.63	0.820
Sex (male), *n* (%)	94 (85.5)	36 (92.3)	58 (81.7)	0.131
HBsAg-positive (+), *n* (%)	74 (67.3)	28 (71.8)	46 (64.8)	0.454
DM, *n* (%)	29 (26.7)	15 (38.5)	14 (19.7)	0.033
HTN, *n* (%)	49 (44.5)	17 (43.6)	32 (44.4)	0.881
LC, *n* (%)	65 (59.1)	25 (61)	40 (56.3)	0.248
Child–Pugh class (B/A)	9/101	2/37	7/64	0.386
Tumor size (≥5 cm/<5 cm)	25/85	11/28	14/57	0.310
Tumor number	1.24 ± 0.54	1.13 ± 0.34	1.30 ± 0.62	0.120
PVI	2 (1.8)	1 (2.6)	1 (1.3)	0.664
WBC	6285.5 ± 3075.6	6335.9 ± 3271.5	6257.8 ± 2986.1	0.899
Platelet	153.38 ± 62.08	159.46 ± 70.68	150.04 ± 57.08	0.449
Serum ALT (IU/L)	34.81 ± 20.95	36.96 ± 19.05	33.63 ± 21.98	0.430
AFP > 400, *n* (%)	19 (17.3)	4 (21.1)	15 (21.1)	0.149

ALT, alanine transaminase; AFP, alpha-fetoprotein; DM, diabetes mellitus; HBsAg, hepatitis B virus surface antigen; HTN, hypertension; LC, liver cirrhosis; PVI, portal vein invasion; WBC, white blood cell.

**Table 2:** Univariate and multivariate cox analysis for mortality following surgical resection.

Variables	Univariate Analysis			Multivariate Analysis		
	HR	95% CI	*p*-Value	HR	95% CI	*p*-Value
Age (years)	0.974	0.929–1.021	0.268			
Sex (male/female)	39.152	0.064–123930.4	0.263			
DM	0.486	0.107–2.210	0.351			
HTN	0.546	0.171–1.749	0.308			
HBsAg-positive (+)	0.796	0.266–2.383	0.684			
Disease status (LC/CHB)	0.983	0.340–2.842	0.975			
Child–Pugh class (B/A)	0.929	0.121–7.147	0.943			
Tumor size (≥5 cm/<5 cm)	3.147	1.053–9.403	0.040	4.060	1.322–12.473	0.014
Tumor number (single vs. multiple)	1.986	0.640–6.168	0.235			
Platelet	1.000	0.991–1.009	0.976			
Serum ALT (IU/L)	1.002	0.979–1.026	0.873			
AFP > 400	1.566	0.430–5.705	0.497			
PrP^C^ (high/low)	4.161	0.927–18.667	0.063	5.246	1.140–24.131	0.033

ALT, alanine transaminase; AFP, alpha-fetoprotein; CHB, chronic hepatitis B; CI, confidence interval; DM, diabetes mellitus; HR, hazard ratio; HTN, hypertension; LC, liver cirrhosis; PrP^C^, cellular prion protein.

**Table 3:** Univariate and multivariate logistic analysis for 1-year recurrence following surgical resection.

Variables	Univariate Analysis			Multivariate Analysis		
	OR	95% CI	*p*-Value	OR	95% CI	*p*-Value
Age (years)	0.971	0.934–1.009	0.136			
Sex (male/female)	0.479	0.156–1.473	0.199			
DM	0.559	0.190–1.647	0.291			
HTN	0.431	0.170–1.095	0.077	0.364	0.131–1.011	0.052
HBsAg-positive (+)	1.209	0.471–3.105	0.693			
Disease status (LC/CHB)	1.009	0.417–2.442	0.984			
Child–Pugh class (B/A)	2.713	0.673–10.943	0.161			
Tumor size (≥5 cm/<5 cm)	2.667	1.020–6.969	0.045	3.212	1.043–9.895	0.042
Tumor number (single vs. multiple)	1.295	0.446–3.760	0.634			
Platelet	0.996	0.989–1.003	0.264			
Serum ALT (IU/L)	0.996	0.974–1.018	0.720			
AFP > 400	3.650	1.293–10.304	0.014	2.134	0.692–6.580	0.187
PrP^C^ (high/low)	3.053	1.052–8.857	0.040	3.540	1.119–11.201	0.031

ALT, alanine transaminase; AFP, alpha-fetoprotein; CHB, chronic hepatitis B; CI, confidence interval; DM, diabetes mellitus; HR, hazard ratio; HTN, hypertension; LC, liver cirrhosis; PrP^C^, cellular prion protein.

## Data Availability

No new data were created or analyzed in this study. Data sharing is not applicable to this article.
